# Fecal carriage and molecular characterization of carbapenem-resistant *Enterobacteriaceae* from hospitalized children in a tertiary hospital of Shandong, China

**DOI:** 10.3389/fmicb.2025.1542207

**Published:** 2025-02-18

**Authors:** Xia Deng, Shuyun Wang, Peibin Hou, Na Sun, Ying Yang, Qian Zeng, Juan Wang, Chunping Wang, Xin Lv, Wenqiang Zhang, Ruyue Fan

**Affiliations:** ^1^School of Public Healthy, Shandong Second Medical University, Weifang, China; ^2^Clinical Laboratory, Jinan Children's Hospital, Children's Hospital Affiliated to Shandong University, Jinan, China; ^3^Shandong Center for Disease Control and Prevention, Jinan, China; ^4^Shandong Provincial Key Laboratory of Infectious Diseases Control and Prevention, Jinan, China

**Keywords:** carbapenem-resistant *Enterobacteriaceae*, hospitalized children, antimicrobial drug resistance, carbapenemase genes, multilocus sequence typing

## Abstract

**Background:**

The prevalence of carbapenem-resistant *Enterobacteriaceae* (CRE) has emerged as a serious public health problem worldwide, and the data on the fecal carriage of CRE strains in hospitalized children remain limited. This study aimed to investigate the molecular characteristics of intestinal colonization of CRE in hospitalized children in Shandong, China.

**Methods:**

A retrospective study was conducted from August to November 2023. Antimicrobial susceptibility testing was performed by the broth microdilution method. Carbapenemase genes, drug resistance genes, and plasmid replicon types were detected using multiplex real-time PCR and whole-genome sequencing. Multilocus sequence typing (MLST) was used to determine the genetic relationships between strains.

**Results:**

A total of 20 CRE isolates were identified from 432 fecal samples, with a fecal carriage rate of 4.6%. The CRE isolates predominantly consisted of *Escherichia coli* (*E. coli*, *n* = 13) and *Klebsiella strains* (*n* = 6). CRE isolates showed a high resistance rate of 90–100% to seven *β*-lactam antibiotics. Resistance rates for other antibiotics such as trimethoprim-sulfamethoxazole, tetracycline, azithromycin, ciprofloxacin, chloramphenicol, nalidixic acid, and streptomycin were 90, 85, 85, 80, 75, 75, and 75%, respectively. CRE isolates showed low resistance to amikacin (20%), and none of the isolates were resistant to tigecycline. Additionally, the multidrug resistance rate of CRE isolates was 95%. All CRE strains carried sulfonamide antibiotic and *β*-lactamase resistance genes, of which the most common β-lactamase resistance genes were *bla*_NDM-1_ (*n* = 9), *bla*_NDM-5_ (*n* = 7) and *bla*_OXA-1_ (*n* = 7). Resistance genes to tetracycline and macrolide antibiotics were also widespread among the strains. The study found that IncFIB and IncFII series plasmids were present in 84 and 42% of the CRE strains, respectively. Additionally, Col, IncFIA, IncC, IncHI2, and IncX series plasmids were also detected. MLST analysis revealed diverse sequence types (STs) among CRE isolates, with ST167 being a common ST among *E. coli* isolates.

**Conclusion:**

This study revealed *bla*_NDM_*E. coli* were the dominant isolates in fecal samples of hospitalized children in Shandong Province, with a broad multidrug resistance to antibiotics, emphasizing that infection control measures need to be taken to limit the spread of these strains.

## Introduction

CRE are defined as *Enterobacteriaceae*, represented by *K. pneumoniae* and *E. coli*, that exhibit resistance to carbapenems and are not susceptible to extended spectrum cephalosporins. Carbapenems are a class of broad-spectrum antibiotics that are normally used as the last resort to treat serious gram-negative bacterial infections. The data from the World Health Organization (WHO) showed that CRE has been identified as one of the bacteria for which new antibiotics are urgently needed (Tacconelli et al., 2018). CRE has spread globally, with widespread distribution reported in countries across Europe, Asia, Africa, and the Americas, posing a significant threat to human health (Li Y. et al., 2024). In recent years, the prevalence of CRE in China has gradually increased. According to China Antimicrobial Surveillance Network (CHINET) data,^1^ the resistance rate of *K. pneumoniae* to carbapenems has significantly increased from 2.9% in 2005 to 26% in 2023, with this proportion still on the rise. A previous study indicated that the carriage rate of CRE in healthy individuals in Shandong province, China, increased from 2.4% in 2015 to 13.4% in 2017 (Chen B. et al., 2022).

This growing trend of CRE infections poses a global threat to public health, especially in hospitalized children. Due to immature in immune system development and the limited use of antibiotics in clinical treatment, children are more easily at greater risk from CRE infections. Studies conducted on CRE within the setting of children’s hospitals have mostly focused on intensive care units (ICUs). Studies have reported a colonization rate of CRE up to 19.5% in pediatric intensive care units (PICUs) (Liu P. et al., 2022; Krupanandan et al., 2023), with a 30-day mortality rate of 50% in children infected with CRE in PICUs. In Vietnam, 337 of 941 cases (35.8%) during the CRE screening performed on pediatric ICU admissions and cohort care were positive for CRE (Garpvall et al., 2021). However, there has been relatively limited studies that focus on noncritically ill hospitalized children. In three hospitals in southern China, 158 of 4,033 screened fecal samples (3.92%) from pediatric children were CRE positive (Xiong et al., 2023). A tertiary pediatric hospital in Shanghai, China collected 880 fecal samples for screening tests. From these, 32 non-duplicate fecal samples from 32 children were identified, each containing a strain of CRE, resulting in a carriage rate of 3.6% (Pan et al., 2019). The risks of CRE infections to noncritically ill hospitalized children should not be ignored.

Carbapenemase is the main resistance mechanism of CRE. This type of enzyme belongs to a special type of beta-lactamase that can effectively break down carbapenem antibiotics, leading to bacterial resistance. The main types of carbapenemases include *K. pneumoniae* carbapenemase (KPC), New Delhi metallo-*β*-lactamase (NDM), and Verona Integron-Encoded metallo-β-lactamase (VIM). Among these types, *bla*_NDM_ is particularly prevalent in children (Fu et al., 2022), suggesting that we need to pay special attention to this type of resistance mechanism in antibiotic treatment strategies for children. Numerous variants of the NDM, such as NDM-1 (the primary variant) and its derivatives *bla*_NDM-2_, *bla*_NDM-3_, *bla*_NDM-4_, and *bla*_NDM-5_ (secondary variants), have been identified globally (Farooq and Khan, 2023). The genes encoding *bla*_NDM-1_ are typically located on plasmids, which facilitates their easy transfer among microorganisms via horizontal gene transfer (Findlay et al., 2021). The transmission of *bla*_NDM-1_ producing strains can occur through cross-contamination during food preparation or via bodily fluids, a process that can happen in both community and hospital settings (Zhai et al., 2021). The presence of these carbapenemase genes in the intestinal microbiota of children is particularly concerning with children serving as carriers and being vulnerable to infection (Xu et al., 2023). The colonization of CRE in the intestines of children might lead to the spread of resistant bacteria within the community and hospitals, posing a risk to other susceptible children. However, relatively little is known about the prevalence of CRE colonization and carbapenemase genes in the intestines of children in northern China.

In this study, we isolated and identified CRE strains in fecal samples from hospitalized children in Shandong, northern China, and investigated the molecular epidemiology, antimicrobial drug resistance, and resistance mechanisms of CRE isolates. Genomic characterization indicated the genetic diversity of drug resistance genes in intestinal colonization of CRE isolates, and antimicrobial drug resistance profiles might have become prevalent among CRE strains.

## Methods and materials

### Sample collection

Fecal samples were collected from hospitalized children (aged ≤14 years) at the Clinical Laboratory of Children’s Hospital Affiliated with Shandong University, a major pediatric hospital in Shandong Province, China. Each fecal sample was placed in a separate sterile tube and transported to the laboratory at the Shandong Center for Disease Control and Prevention under refrigeration at 4°C.

### Isolation of CRE strains

Samples were homogenized with 1 mL of 0.9% saline and plated on MacConkey agar containing 2 μg/mL meropenem. Plates were incubated at 37°C for 18–24 h. Colonies indicative of CRE growth were subcultured on brain heart infusion agar and incubated under the same conditions. The identification of CRE strains was preliminary performed using the MALDI Biotyper sirius IVD (Bruker Daltonics GmbH & Co. KG, Germany) and the VITEK^®^ 2 Compact system (BioMérieux, France). CRE isolates were stored in 40% glycerol at −80°C for further analysis.

### DNA extraction and carbapenemase genes screening by multiplex real-time PCR

To screen for carbapenemase genes in CRE isolates, multiplex real-time PCR was performed on identified CRE strains as previously described (Monteiro et al., 2012). DNA was extracted from CRE isolates using the Endo-Free Plasmid Mini Kit I (Omega Bio-Tek, United States). DNA concentration was standardized to 10 ng/μL. Carbapenemase genes were detected using multiplex real-time PCR with primers as detailed in Table 1. The PCR mixture included 12.5 μL of 2× HRM PCR master mix, primers at optimized concentrations (IMP-F and IMP-R at 1.2 μM, others at 0.2 μM), and 1 μL of DNA. PCR was conducted on the QuantStudio 7 Flex (Thermo Fisher Scientific, USA) with the following cycle: initial denaturation at 95°C for 5 min, 35 cycles of denaturation at 95°C for 20 s, annealing at 55°C for 45 s, and extension at 72°C for 30 s. A melt curve from 65°C to 95°C confirmed amplicon specificity.

**Table 1:** Primers used in this study.

Target	Primer name	Sequence (5′ –3′)	Amplicon size (bp)	Primer concentration (mM)	Tm
*bla*_KPC_	KPC-F	TCGCTAAACTCGAACAGG	785	0.2	91.6
	KPC-R	TTACTGCCCGTTGACGCCCAATCC			
*bla*_NDM_	NDM-F	TTGGCCTTGCTGTCCTTG	82	0.2	84
	NDM-R	ACACCAGTGACAATATCACCG			
*bla*_GES_	GES-F	CTATTACTGGCAGGGATCG	594	0.2	88.6
	GES-R	CCTCTCAATGGTGTGGGT			
*bla*_OXA-48_	OXA-48-F	TGTTTTTGGTGGCATCGAT	177	0.2	81.6
	OXA-48-R	GTAAMRATGCTTGGTTCGC			
*bla*_IMP_	IMP-F	GAGTGGCTTAATTCTCRATC	120	1.2	80.1
	IMP-R	AACTAYCCAATAYRTAAC			
*bla*_VIM_	VIM-F	GTTTGGTCGCATATCGCAAC	382	0.2	90.3
	VIM-R	AATGCGCAGCACCAGGATAG			

### Antimicrobial susceptibility testing

Antimicrobial susceptibility testing was conducted using the Customized AST plate CHNENF and Sensititre ARIS 2X (Thermo Fisher Scientific, United States) according to the manufacturer’s instructions. The plate includes 17 antimicrobial agents: *β*-lactam antibiotics including ceftazidime/avibactam (CZA), ertapenem (ETP), cefotaxime ampicillin (CAZ), cefotaxime (CTX), ampicillin (AMP), ampicillin/sulbactam (AMS), meropenem (MEM); tetracycline antibiotics including tigecycline (TIG) and tetracycline (TET); fluoroquinolone drugs including ciprofloxacin (CIP) and nalidixic acid (NAL); aminoglycoside antibiotics including streptomycin (STR) and amikacin (AMI); and azithromycin (AZM), trimethoprim/sulfamethoxazole (SXT), polymyxin E (colistin; COL), chloramphenicol (CHL). The strains for quality control were *E. coli* strain ATCC 25922. Interpretation followed the latest CLSI breakpoints (M100-ED32, M45-ED3, NARMS).

### Whole genome sequencing

Genomic DNA was extracted with the TaKaRa MiniBEST Bacterial Genomic DNA Extraction Kit. DNA purity (A260/280 ratio between 1.8 and 2.0) was confirmed via UV spectrophotometry. To obtain the draft genomes of CRE isolates, library preparation and whole-genome sequencing were performed at Novogene Bioinformatics Technology Co., Ltd., Beijing, using the Illumina NovaSeq (PE150) platform. Sequencing data were assembled and analyzed for core genome multilocus sequence typing (cgMLST) clustering through the National Foodborne Disease Molecular Tracing Network (TraNet). Genomic annotation was performed using the Prokka software (version 1.14.6). MLST, resistance genes, virulence genes, and plasmid analysis were conducted using Abricate software in combination with the VFDB, ResFinderand, and Pathogen Watch databases. The virulence genes were mapped to the search tool for retrieval of interacting genes (STRING) to acquire protein–protein interaction (PPI) networks.

### Statistical analysis

Statistical analyses were performed using SPSS software 22 (SPSS Inc., Chicago, IL, United States). The *χ*^2^ test was used to evaluate differences in gender data, and the Fisher exact test was used to evaluate differences in age and hospital department data. *p* < 0.05 was considered a statistically significant difference.

## Results

### Samples collection and CRE isolation

During the study period, we collected 434 fecal samples from hospitalized children at the Clinical Laboratory of Children’s Hospital Affiliated to Shandong University. The mean age of the participants was 5.19 ± 3.53 years (range: 1 day to 14 years), comprising 271 males (62.4%) and 163 females (37.6%), though this difference was not statistically significant (*p* > 0.05). We successfully isolated CRE from 20 non-repetitive samples, representing an overall positivity rate of 4.6%. The 20 isolated strains included *E. coli* (*n* = 13), *Klebsiella pneumoniae* (*K. pneumoniae, n* = 2)*, Klebsiella* var*iicola* (*K. variicola*, *n* = 2), *Klebsiella pasteurii* (*K. pasteurii*, *n* = 1), *Klebsiella aerogenes* (*K. aerogenes*, *n* = 1), and *Raoultella ornithinolytica* (*R. ornithinolytica*, *n* = 1). The age distribution of CRE-positive samples revealed the highest incidence in the 0 ~ 1 year group (8.5%, 6/71), with no cases detected in the 9 ~ 14 year age group (*p* > 0.05). Department-wise, the highest incidence was observed in neonatology and pediatric surgery (18.5%, 5/27), significantly higher than in other departments, such as Gastroenterology, Neuroendocrinology, and Otolaryngology (*p* < 0.05). These 20 CRE isolates were subjected to whole-genome sequencing. In brief, the size of the genomes ranged from 4.86 to 6.52 million base pairs (Mbp), with an approximate GC content of 52.7%. The genomic features of CRE isolates are summarized in Table 2. Sequences were deposited in NCBI GenBank under project number PRJNA1074525.

**Table 2:** Genome size and features of genomes in sequenced strains in this study.

No.	Genome size (Mb)	G + C (%)	Coverage (fold)	Contigs (*n*)	CDS (*n*)	Genbank accession
E.1	4.92	50.45%	293	105	4,192	SRR27920372
E.2	5.02	50.69%	325	132	4,248	SRR27920385
E.3	4.86	50.59%	267	156	4,134	SRR27920384
E.4	5.04	50.70%	289	79	4,314	SRR27920383
E.5	5.41	50.31%	248	141	4,465	SRR27920382
E.6	4.86	50.54%	225	96	4,150	SRR27920381
E.7	5.23	50.40%	341	107	4,397	SRR27920380
E.8	5.00	50.79%	308	107	4,895	SRR27920375
E.9	4.92	50.79%	313	103	4,066	SRR27920373
E.10	5.07	50.47%	317	108	4,279	SRR27920389
E.11	5.16	50.38%	367	108	4,254	SRR27920388
E.12	5.01	50.69%	362	135	4,247	SRR27920386
E.13	5.02	50.75%	360	122	4,670	SRR27920387
K.1	5.52	57.01%	331	38	5,200	SRR27920378
K.2	5.38	57.09%	332	60	5,204	SRR27920379
K.3	5.62	56.68%	349	38	5,524	SRR27920390
K.4	5.65	56.57%	352	44	5,553	SRR27920377
K.5	6.52	54.81%	394	64	6,196	SRR27920391
K.6	4.91	55.26%	300	24	4,615	SRR27920374
R.1	5.52	55.28%	351	35	5,512	SRR27920376

### Screening of carbapenemase genes

We initially screened carbapenemase genes using multiplex real-time PCR. Carbapenemase genes were detected in all CRE isolates, with *bla*_NDM_ being the most prevalent (95%, 19/20), followed by *bla*_GES_ (5%, 1/20). Other carbapenemase genes such as *bla*_IPM_*, bla*_OXA-48_*, bla*_VIM_, and *bla*_KPC_ were not found in our screening.

### Antimicrobial susceptibility

The results of the antimicrobial susceptibility tests in CRE isolates were shown in Table 3, and the number of antibiotic-susceptible, −intermediate, and -resistant isolates were shown in Table 4. The resistance profile against *β*-lactam antibiotics of CRE isolates was 90–100%, followed by SXT (90%), AMZ (85%), TET (85%), and CIP (80%). The resistance rates to CHL, NAL, and STR were 75%, respectively. CRE isolates showed low resistance to AMI (20%). None of the isolates were resistant to TIG. The intermediate and resistant rates of isolates to COL were 80 and 20%, respectively (Table 4). The rate of multidrug resistance of CRE isolates was 95%.

**Table 3:** Minimal inhibitory concentration values of CRE isolates in fecal samples from hospitalized children.

NO.	Species	MIC (μg/ml)																
		CZA	ETP	MEM	CAZ	CTX	AMP	AMS	CHL	SXT	COL	AZM	STR	AMI	TIG	TET	CIP	NAL
E.1	*E. coli*	>8	>8	>2	>16	>16	>32	>32	>32	>8	<=0.25	>64	>32	<=4	<=0.25	>16	>2	>32
E.2	*E. coli*	0.5	8	2	>16	>16	>32	>32	>32	>8	<=0.25	>64	>32	<=4	<=0.25	8	>2	>32
E.3	*E. coli*	>8	8	>2	>16	>16	>32	>32	>32	>8	8	16	>32	<=4	1	>16	2	>32
E.4	*E. coli*	>8	>8	>2	>16	>16	>32	>32	>32	>8	0.5	>64	>32	64	<=0.25	>16	>2	>32
E.5	*E. coli*	>8	>8	>2	>16	>16	>32	>32	>32	>8	8	>64	>32	>64	<=0.25	>16	>2	>32
E.6	*E. coli*	>8	>8	>2	>16	>16	>32	>32	>32	>8	<=0.25	64	>32	<=4	0.5	>16	>2	>32
E.7	*E. coli*	>8	>8	>2	>16	>16	>32	>32	>32	>8	1	>64	>32	8	<=0.25	>16	>2	>32
E.8	*E. coli*	>8	>8	>2	>16	>16	>32	>32	>32	>8	<=0.25	>64	>32	<=4	<=0.25	>16	>2	>32
E.9	*E. coli*	>8	8	>2	>16	>16	>32	>32	16	<=0.5	<=0.25	64	8	<=4	1	4	0.06	4
E.10	*E. coli*	>8	>8	>2	>16	>16	>32	>32	8	>8	<=0.25	32	16	<=4	<=0.25	>16	>2	>32
E.11	*E. coli*	>8	>8	>2	>16	>16	>32	>32	>32	>8	<=0.25	>64	>32	<=4	<=0.25	>16	>2	>32
E.12	*E. coli*	>8	>8	>2	>16	>16	>32	>32	>32	>8	<=0.25	16	>32	<=4	<=0.25	>16	0.25	<=4
E.13	*E. coli*	>8	>8	>2	>16	>16	>32	>32	<=4	>8	<=0.25	8	<=8	<=4	0.5	>16	0.5	8
K.1	*K. pneumoniae*	>8	>8	>2	>16	>16	>32	>32	>32	>8	<=0.25	>64	>32	<=4	<=0.25	>16	>2	>32
K.2	*K. pneumoniae*	>8	>8	>2	>16	>16	>32	>32	>32	>8	4	>64	>32	64	1	>16	>2	>32
K.3	*K. variicola*	>8	>8	>2	>16	>16	>32	>32	4	>8	<=0.25	8	8	<=4	0.5	>16	0.5	8
K.4	*K. variicola*	>8	>8	>2	>16	>16	>32	>32	8	>8	0.5	>64	<=8	<=4	0.5	2	>2	16
K.5	*K. pasteurii*	>8	8	>2	>16	>16	>32	>32	>32	>8	8	>64	>32	>64	<=0.25	>16	>2	>32
K.6	*K. aerogenes*	<=0.25	>8	>2	>16	>16	>32	>32	>32	>8	<=0.25	64	32	<=4	0.5	16	>2	>32
R.1	*R. ornithinolytica*	>8	>8	>2	>16	>16	>32	>32	>32	>8	<=0.25	>64	>32	<=4	<=0.25	>16	>2	>32

CZA, ceftazidime-avibactam; ETP, ertapenem; MEM, meropenem; CAZ, cefotaxime ampicillin; CTX, cefotaxime; AMP, ampicillin; AMS, ampicillin-sulbactam; CHL, chloramphenicol; SXT, trimethoprim-sulfamethoxazole; COL, colistin; AZM, azithromycin; STR, streptomycin; AMI, amikacin; TIG, tigecycline; TET, tetracycline; CIP, ciprofloxacin; NAL, nalidixic acid.

**Table 4:** Number of antibiotic-susceptible, -intermediate, and -resistant isolates.

Antibiotics	Susceptible isolates (%)	Intermediate isolates (%)	Resistant isolates (%)
Amikacin	16 (80%)	0 (0%)	4 (20%)
Ampicillin	0 (0%)	0 (0%)	20 (100%)
Ampicillin-Sulbactam	0 (0%)	0 (0%)	20 (100%)
Azithromycin	3 (15%)	-	17 (85%)
Cefotaxime	0 (0%)	0 (0%)	20 (100%)
Ceftazidime	0 (0%)	0 (0%)	20 (100%)
Ceftazidime-Avibactam	2 (10%)	-	18 (90%)
Chloramphenicol	4 (20%)	1 (5%)	15 (75%)
Ciprofloxacin	2 (10%)	2 (10%)	16 (80%)
Colistin	-	16 (80%)	4 (20%)
Ertapenem	0 (0%)	0 (0%)	20 (100%)
Meropenem	0 (0%)	1 (5%)	19 (95%)
Nalidixicacid	5 (25%)	-	15 (75%)
Streptomycin	5 (25%)	-	15 (75%)
Tetracycline	2 (10%)	1 (5%)	17 (85%)
Tigecycline	20 (100%)	0 (0%)	0 (0%)
Trimethoprim-sulfamethoxazole	1 (10%)	-	19 (90%)

### STs patterns

**Figure 1:**
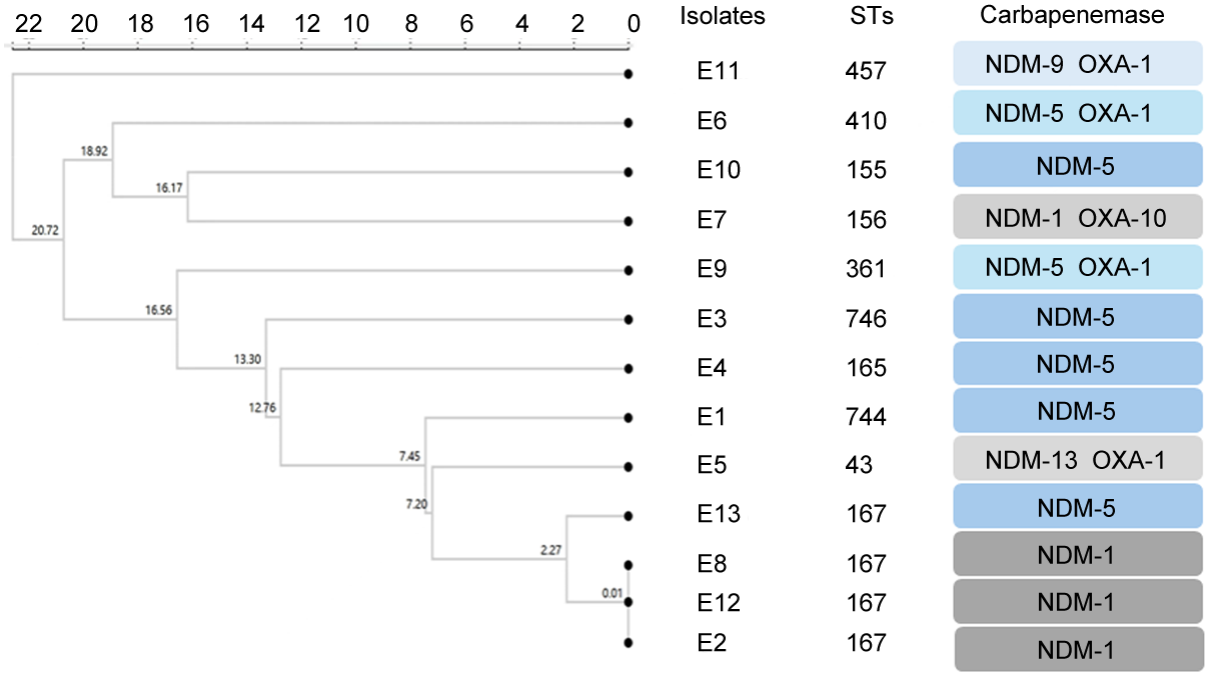
Clustering tree constructed using UPGMA algorithm for carbapenem-resistant *E. coli*. Annotation information includes MLST typing, CRE genotype.

The results of MLST typing of carbapenem-resistant *E. coli* isolates were shown in Figure 1. MLST revealed 10 sequence types among the 13 carbapenem-resistant *E. coli* isolates, with ST167 being the most common. Notably, three patients were carried by the carbapenem-resistant *E. coli* isolates with the same STs patterns and carbapenemases. Among the *Klebsiella* strains, STs included ST37 and ST462 for *K. pneumonia* K.2 *and* K.1, ST3200 for *K.* var*iicola*, ST4 for *K. aerogenes*, and ST*ff57 for *K. pasteurii*.

### Characteristics of antimicrobial resistance genes

**Figure 2:**
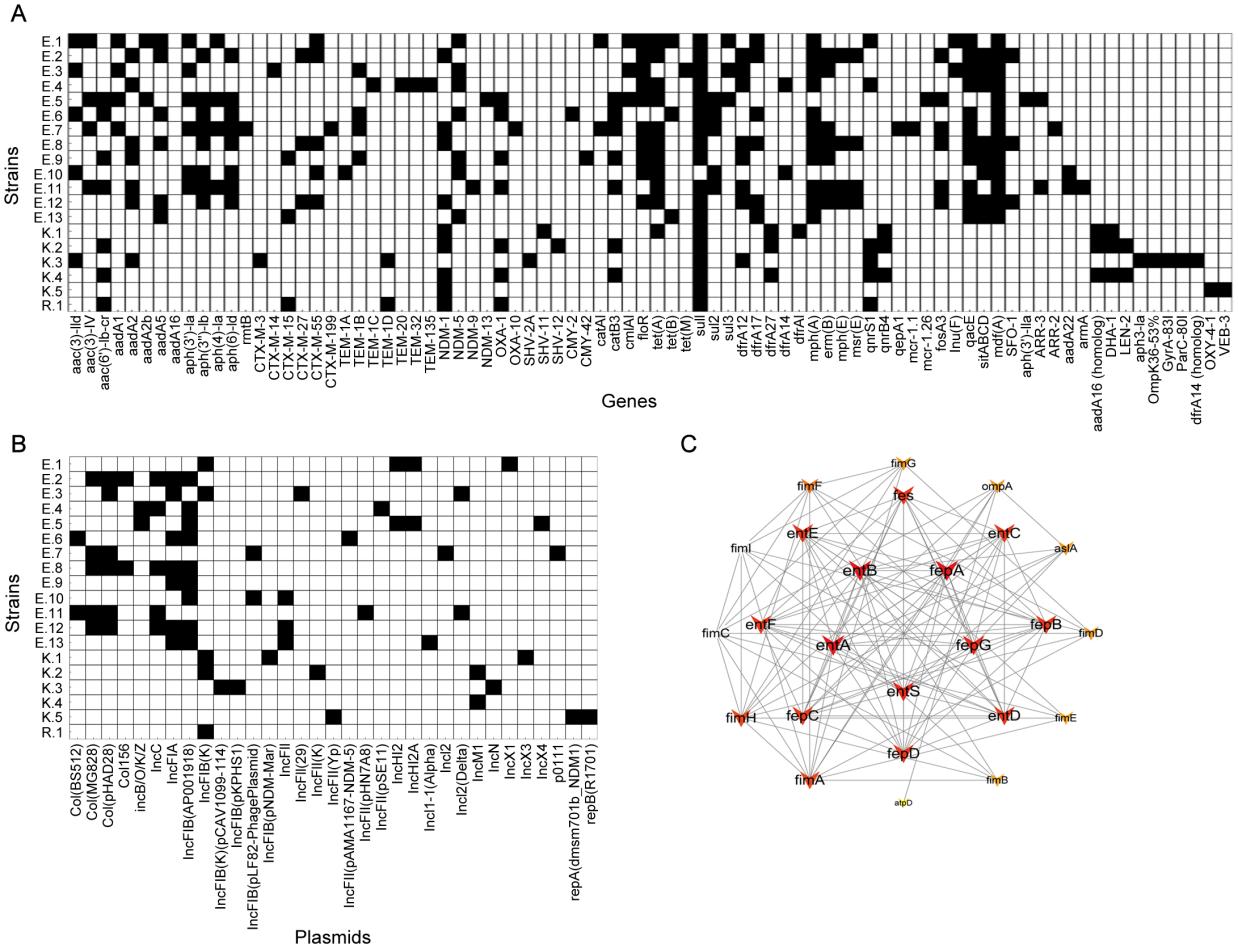
A heat-map summary of the sources. **(A)** A heat map of antimicrobial resistance genes. **(B)** A heat map of plasmid replicon types. Black squares shown indicated the feature of antimicrobial resistance profiles and the plasmid replicon types present in CRE isolates. **(C)** Virulence gene network interaction diagram of CRE isolates.

All CRE strains tested harbored sulfonamide antibiotic resistance genes and *β*-lactamase genes (Figure 2A). Among them, β-lactamase genes show a high degree of diversity, and common drug resistance genes include *bla*_NDM-1_ (*n* = 9), *bla*_NDM-5_ (*n* = 7) and *bla*_OXA-1_ (*n* = 7), followed by *bla*_CTX-M-55_ (*n* = 5), *bla*_CTX-M-55TEM-1B_ (*n* = 4), as well as *bla*_CTX-M-15_, *bla*_SFO-1_, *bla*_CTX-M-27_ and *bla*_DHA-1_ (*n* = 5, respectively). In terms of sulfonamide antibiotic resistance genes, most strains harbored *sul*1 gene (*n* = 18), followed by *sul*2 (*n* = 5) and *sul*3 (*n* = 3). Among the TET resistance genes, *tet*A detected in 11 isolates, *tet*B detected in 3 isolates, and *tet*M detected in one isolate. Among the macrolide antibiotic resistance genes, *mph*A and *erm*B detected in 11 and 7 isolates, respectively, and *msr*E and *mph*E detected in 4 isolates. Two *E. coli* isolates (E.5 and E.7) were found to carry the *mcr* gene variants (*mcr-1.26* and *mcr-1.1*).

In addition, this study also found other important resistance genes: 74% of the strains carried TET resistance genes, 79% of the strains carried dihydrofolate reductase genes, of which *dfr*A12 *was* detected in 8 isolates, *dfr*A17 *was* detected in 6 isolates, 68% of the strains carried multidrug resistance efflux pump genes, of which *mdf*A *was* detected in 13 isolates, and 58% of the strains carried antiseptic resistance genes and macrolide antibiotics, of which *qnrS1 was* detected in 5 isolates, *qnrB4 was* detected in 3 isolates, 53% of the strains carried *fos*A3 fenfenicol resistance gene (*n* = 5), 47% of the strains carried *cat*B3 CHL resistance gene (*n* = 6), 32% of the strains carried quinolone resistance genes, and 26% of the strains carried fosfomycin resistance genes.

### Characteristics of plasmid replicon types

In the plasmid analysis of CRE isolates, a distribution of multiple plasmid types was found (Figure 2B). IncFIB series plasmids were detected in 84% of strains, of which IncFIB (K) was detected in 5 isolates and IncFIB (AP001918) was detected in 8 isolates, which were the most common plasmid types. IncFIB (pLF82-PhagePlasmid), IncFIB (pKPHS1), IncFIB(K) (pCAV1099-114) and IncFIB (pNDM-Mar) were detected in one strain, respectively. IncFII series plasmids were found in 42% of the strains, including IncFII (pHN7A8), IncFII (29), IncFII (pSE11), IncFII (pAMA1167-NDM-5) and IncFII (K). IncI series plasmids were detected in 21% of strains, with IncI2 (Delta) found in two strains, IncI1-I (Alpha) and IncI2 detected in one strains, respectively. Col series plasmids were found in 37% of strains, including Col(pHAD28) (*n* = 5) and Col(MG828) (*n* = 5), and Col(BS512) (*n* = 2) and Col156 (*n* = 2). In addition, the carriages rates of IncFIA series, IncC series, IncHI2 series and IncX series plasmids of CRE isolates were 37, 26, 11 and 16%, respectively.

### Characteristics of virulence genes

*For the examination of the virulence factor of CRE isolates, entA*, *entB*, *entC*, *entD*, *entE*, *entF*, *entS*, *fepA*, *fepB*, *fepC*, *fepD*, *fepG, fes*, *espR1*, and *ompA* were *identified* in all CRE isolates. The STRING database-based protein–protein interaction (PPI) network revealed that entA, entB, entS, fepA, and fepG coded by the *entA*, *entB*, *entS*, *fepA*, and *fepG* genes formed a core group in the *E. coli* virulence protein interaction network (Figure 2C).

## Discussion

This study investigated the carriage of CRE in fecal samples of hospitalized children in Shandong Province and found a CRE detection rate of 4.6% in the children’s feces. This rate was comparable to previous report, such as a 4.5% CRE positivity rate in rectal swabs from children in the United Kingdom in 2012 (Drew et al., 2013), and a 5.2% detection rate in the United States (Logan et al., 2015). In China, a previous study revealed the CRE positivity rate for pediatric inpatients in Southern China is 3.92% (Xiong et al., 2023), while in Shanghai, the carriage rate for outpatient children was 3.6% in 2016 (Pan et al., 2019), and 8.6% for hospitalized children in 2019 (Xu et al., 2020). Our results indicated that the carriage rate of CRE in children in Shandong, northern China was not significantly higher than in these regions, reflecting a moderate CRE burden in our pediatric population comparable to both Western and other regional Chinese settings.

Regarding age grouping, there was no significant difference was found in the detection rate of CRE in children in this study. The overall prevalence of CRE in children in China was reported is 6.4%, with 8.8% in the neonatal period, 7.3% in infancy, 3.8% in toddlerhood, 4.0% in the preschool period, 4.7% in school age, and 7.4% in adolescence (Guo et al., 2018). Notably, our study found that the detection rate in neonatology and neonatal surgery was significantly higher than in other departments. This finding was consistent with a study from the Asian region in 2023, which indicated that infants under one month of age had a higher rate of infection than other children (Yen et al., 2023). This suggests that neonates, particularly those requiring surgical intervention, are at increased susceptibility to CRE infections, underscoring the need for stringent infection control measures in these high-risk areas.

This study conducted a detailed analysis of the antimicrobial drug resistance profiles of CRE strains. The findings of this study were consistent with previous reports, confirming that CRE strains were commonly resistant to many classes of antibiotics (Wang et al., 2018), which provided an important basis for future prevention and treatment strategies and guidance on antibiotic use. The higher multidrug resistance rate observed in this study further highlighted the major challenges in clinical treatment. It was particularly noteworthy that the resistance rate to *β*-lactam antibiotics was as high as 90–100%, which reflected the widespread clinical use of this class of drugs and the corresponding resistance pressure. In addition, the resistance rates to SXT and TET were as high as 95 and 85% respectively, highlighting the prevalence of CRE isolates resistant to these commonly used antimicrobials. Resistance rates to aminoglycoside antibiotics in particular were also detected to be higher, posing a serious challenge to treatment options for pediatric patients, as some key antibiotics had limited use in children. For instance, fluoroquinolones were unsuitable for children and adolescents because of potential damage to joint cartilage and effects on bone growth (Patel and Goldman, 2016); and aminoglycosides, although commonly used, could cause ototoxicity and nephrotoxicity if overused (Le et al., 2023); Tetracycline antibiotics may affect bone and tooth development in children (Sapadin and Fleischmajer, 2006). At the same time, there were some differences compared to the previous report (Zhou et al., 2020), which found higher susceptibility rates to CZA and COL. In this study, four CRE isolates showed resistance to COL. Among them, a novel colistin-resistance gene variant, *mcr-1.26,* was found in one COL-resistant isolate. Besides, one *E. coli* isolate was found to carry the *mcr-1.1* gene, with an intermediate resistance phenotype to COL. However, mutations in chromosomal resistance genes (*mgrB*, *pmrAB*, *phoPQ*, and *crrB*), as well as plasmid-mediated colistin-resistance genes (*mcr* genes) and variants, were not found in the three isolates that were resistant to colistin. This might be due to the presence of undetected drug resistance gene subtypes or mutations not covered by the current sequencing methods in strains.

Our results showed that CRE strains generally carried multiple drug resistance genes. In particular, *β*-lactamase antibiotic resistance genes such as *bla*_NDM-1_*, bla*_NDM-5_, and *bla*_OXA-1_ are ubiquitous among strains, and the high prevalence of these genes was consistent with CRE discovered in many countries worldwide (Wu et al., 2019). In addition, a variety of types of resistance genes were discovered in our study, including the sulfa antibiotic resistance genes *sul*1*, sul2* and *sul3,* and the TET resistance gene *tet*A. The presence of these genes reflected the bacterial response to the environment. Adaptability to antibiotic selection pressures. The prevalence of macrolide antibiotic resistance genes such as *mph*A and *erm*B also indicated the difficulties that these antibiotics might encounter in clinical practice (Cao et al., 2010). For fluoroquinolones and other broad-spectrum antibiotics, we observed that 32% of strains carried quinolone resistance genes, a finding consistent with the global trend of increasing quinolone resistance (Aldred et al., 2014). The increase in such resistance was associated with a high frequency of use, inappropriate use and widespread use in agriculture (Samreen et al., 2021). It was worth noting that the ubiquity of multidrug resistance efflux pump genes such as *mdf*A illustrates the ability of CRE strains to enhance their drug resistance through multiple mechanisms (Zhou et al., 2022). This type of efflux pump could pump a variety of antibiotics out of the cell, effectively reducing the internal antibiotic concentration, thereby reducing the bactericidal or bacteriostatic effect of the antibiotics.

The cluster analysis results of this study showed that three patients carried carbapenem-resistant *E. coli* isolates with ST167, suggesting that dominant strains of this ST type might exist in children’s hospitals. It was worth noting that the ST167 strain had not been reported before 2017, but the latest research showed that this strain was not only found in hospital patients but also in healthy individuals, indicating its possible spread in the population wider than expected (Chen Q. et al., 2022). Globally, *ST167* strains were widely distributed and found in 164 strains in 25 countries, 95 of which carry the bla_NDM_ gene, and *bla_NDM-5_* was the most common. In China, ST167 strains have the highest prevalence, accounting for 37% (Li L. et al., 2024). These data indicated that the prevalence of *ST167* carbapenem-resistant *E. coli* was a public health issue requiring global attention. Moreover, *Klebslella pasteurii* strain isolated was with unique or incompletely defined sequence type ST*ff57 and the type of *Raoultella ornithinolytica* strain cannot be determined. *Raoultella ornithinolytica* was reclassified from the genus *Klebsiella* in 2001, so data on its epidemiology and clinical relevance are not yet complete, and more studies are needed to determine its ST pattern (Hajjar et al., 2020).

In plasmid analysis, we observed that IncFIB and IncFII series plasmids were extremely prevalent in CRE strains, suggesting that these plasmids might be the main vectors of horizontal transmission of resistance genes. Especially for the IncFIB family, its frequent detection in multiple strains highlighted its central role in the spread of antibiotic resistance. According to existing studies, the high prevalence of plasmids such as IncFIB, IncFII, IncR and IncFIA in CRE further indicated their importance in the spread of multidrug resistance (Livorsi et al., 2018). These findings highlighted the role of specific plasmids in antibiotic resistance and provided important information for further research and countermeasures. Although the IncX3 plasmid showed high prevalence in environmental samples from Shandong and children’s samples from Shanghai (Xu et al., 2020; Guo et al., 2023), the detection rate of IncX3 was lower in children’s samples from Shandong, while the IncFIB plasmid appeared to be particularly common. This difference might stem from the different compositions of the microbial communities. In our study, the main CRE strain isolated was carbapenem-resistant *E. coli*. A study on *E. coli* isolates contained a variety of plasmids, mainly IncFIB (AP001918). This plasmid has been confirmed to be associated with resistance to a variety of antibacterial drugs, including *β*-lactas, aminoglycosides, sulfonamides, tetracyclines, etc. (Zhou et al., 2022). These observations highlighted the importance of considering sample type and geography when studying the spread of resistance genes.

Virulence genes involved in the biosynthesis (*entA*, *entB*, *entC*, *entD*, *entE*, *entF*, and *entS*) and uptake (*fepA*, *fepB*, *fepC*, *fepD*, *fepG,* and *fes*) of enterobactin and *ompA* were identified in all isolates. In the virulence protein network of CRE isolates, there were a series of proteins coded by virulence genes that play a core role. Among them, the *ent* genes and the *fep* genes constitute the core of the entire network. The *ent* and *fep* genes are closely related to iron acquisition and are involved in the utilization and transportation of iron, thereby supporting the growth and survival of bacteria in the host environment (Surleac et al., 2020; Casanova-Hampton et al., 2021; Huang et al., 2024). In the outer ring of the virulence protein network, the *fim* genes, *astA*, *ompA*, and *atpD* constitute an indispensable part of the biological function of bacteria. Among them, the weights of *fimH* and *fimA* are relatively large, which may mean that they play a more critical role in the pathogenicity of bacteria. The *fim* genes are responsible for the attachment of bacteria to host cells (Liu Y. et al., 2022; Bachelle et al., 2024). The results revealed that *fimH* and *fimA* might play a role in the attachment and biofilm formation of bacteria, promoting bacterial invasion and drug resistance to the host.

This study filled a research gap on the carriage of CRE among hospitalized children in Shandong Province, northern China, providing preliminary data for understanding the fecal carriage status of CRE in this population. However, this present study has limitations, mainly in sample representativeness and the lack of detailed clinical information. First, only stool sample was collected and detected from each child, and collecting and incorporating data from multiple clinical specimens (blood, urine, and sputum) of hospitalized children may provide additional power to our study. Second, due to a lack of adequate clinical information about hospitalize children, stool samples may be obtained from the children after antibiotic treatment; this may have affected the results. Third, a limitation of this study was the incompleteness of draft genomes generated by Illumina reads and the inability to accurately resolve highly polymorphic regions in genomes, such as resistance genes and mobile genetic elements.

Future research should further delineate population characteristics, especially with more exhaustive observation of the neonatal group, to better understand CRE transmission and infection in different populations. Furthermore, studies with larger sample sizes and additional sequencing and molecular methods for an in-depth analysis of the genome of CRE isolates, especially resistance genes and plasmids, are warranted to confirm these findings and investigate the mechanisms of antimicrobial drug resistance underlying the observed associations.

## Conclusion

In summary, this study demonstrated a major intestinal colonization of *bla*_NDM_ *E. coli* and *Klebsiella* strains among hospitalized children in Shandong, China. All CRE isolates were extensively multidrug-resistant, with multiple resistance genes and plasmid replicon types. To reduce CRE infection and transmission, it is imperative to rigorously implement prevention and control measures in clinical settings, especially in neonatal departments and other high-risk populations. These measures are crucial for reducing infections by multidrug-resistant bacteria and optimizing clinical antimicrobial treatment.

## Data Availability

The datasets presented in the study can be acquired in online repositories. The accession number can be found at: NCBI-PRJNA1074525.

## Footnotes

^1^ www.chinets.com
